# Potential Antitumor Activity of 2-*O*-α-d-Glucopyranosyl-6-*O*-(2-Pentylheptanoyl)-l-Ascorbic Acid

**DOI:** 10.3390/ijms19020535

**Published:** 2018-02-10

**Authors:** Kaori Miura, Misaki Haraguchi, Hideyuki Ito, Akihiro Tai

**Affiliations:** 1 Faculty of Life and Environmental Sciences, Prefectural University of Hiroshima, 5562 Nanatsuka-cho, Shobara, Hiroshima 727-0023, Japan; q531005dd@ed.pu-hiroshima.ac.jp (K.M.); q405088xe@ed.pu-hiroshima.ac.jp (M.H.); 2 Faculty of Health and Welfare Science, Okayama Prefectural University, 111 Kuboki, Soja, Okayama 719-1197, Japan; hito@fhw.oka-pu.ac.jp

**Keywords:** ascorbic acid, stable ascorbic acid derivatives, acyl ascorbic acid, antitumor activity

## Abstract

Intravenous administration of high-dose ascorbic acid (AA) has been reported as a treatment for cancer patients. However, cancer patients with renal failure cannot receive this therapy because high-dose AA infusion can have side effects. To solve this problem, we evaluated the antitumor activity of a lipophilic stable AA derivative, 2-*O*-α-d-glucopyranosyl-6-*O*-(2-pentylheptanoyl)-l-ascorbic acid (6-bOcta-AA-2G). Intravenous administration of 6-bOcta-AA-2G suppressed tumor growth in colon-26 tumor-bearing mice more strongly than did AA, even at 1/10 of the molar amount of AA. Experiments on the biodistribution and clearance of 6-bOcta-AA-2G and its metabolites in tumor-bearing mice showed that 6-bOcta-AA-2G was hydrolyzed to 6-*O*-(2-propylpentanoyl)-l-ascorbic acid (6-bOcta-AA) slowly to yield AA, and the results suggested that this characteristic metabolic pattern is responsible for making the antitumor activity of 6-bOcta-AA-2G stronger than that of AA and that the active form of 6-bOcta-AA-2G showing antitumor activity is 6-bOcta-AA. In in vitro experiments, the oxidized form of 6-bOcta-AA as well as 6-bOcta-AA showed significant cytotoxicity, while the oxidized forms of ascorbic acid showed no cytotoxicity at all, suggesting that the antitumor activity mechanism of 6-bOcta-AA-2G is different from that of AA and that the antitumor activity is due to the reduced and oxidized form of 6-bOcta-AA. The findings suggest that 6-bOcta-AA-2G is a potent candidate as an alternative drug to intravenous high-dose AA.

## 1. Introduction

**Figure 1:**
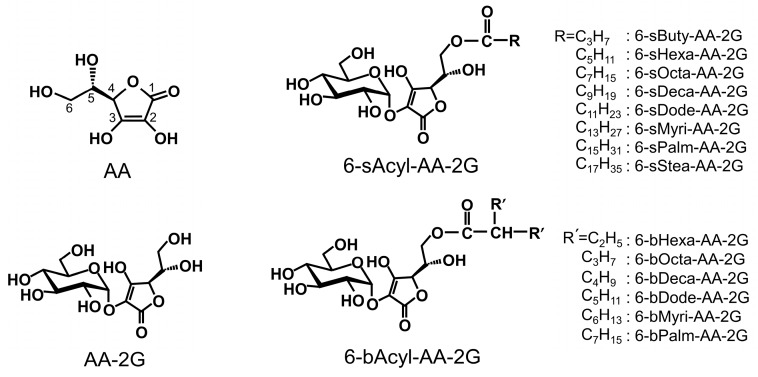
Structures of AA, AA-2G, 6-sAcyl-AA-2G and 6-bAcyl-AA-2G.

Ascorbic acid (AA, Figure 1), known as vitamin C, is a vital nutrient for health. Recently, intravenous high-dose AA has been studied as a treatment for cancer patients [1,2,3,4]. Intravenous infusion of high-dose AA greatly increases plasma AA level to a concentration higher than that achieved by oral administration, and AA exhibits a pro-oxidant activity arising from its reducing property [3,4,5]. AA reduces Fe^3+^ to Fe^2+^, and Fe^2+^ donates an electron to O_2_, ultimately generating reactive oxygen species (ROS) [4,6,7,8]. Normal cells are not injured by ROS due to the activity of antioxidant enzymes such as catalase and glutathione peroxidase. On the other hand, since many cancer cells have lower levels of several antioxidant enzymes than those in normal cells, cell death is induced by injury caused by ROS [9]. More recently, it has been reported that AA enhances the function of TET2, a tumor suppressor [10,11] and that intravenous high-dose AA enhances chemosensitivity of ovarian cancer [12]. However, AA is very unstable in aqueous solutions and is easily decomposed by heat or oxidation. For that reason, an AA infusate requires the addition of a stabilizer and the infusate must be prepared just before use. To solve instability of AA, many stable AA derivatives have been synthesized. 2-*O*-α-d-Glucopyranosyl-l-ascorbic acid (AA-2G, Figure 1) is one of the stable AA derivatives. AA-2G is remarkably stable, even in an aqueous solution, and it rapidly undergoes enzymatic hydrolysis in vivo to supply AA [13,14]. AA-2G exhibits vitamin C activities such as collagen synthesis [15,16] after enzymatic hydrolysis to AA by α-glucosidase. We recently reported that high-dose intravenous AA-2G as well as AA significantly inhibited tumor growth in tumor-bearing mice [17]. The antitumor activity of AA-2G was caused by reactive oxygen species that were generated by AA released by rapid hydrolysis of AA-2G. AA-2G is expected to be an alternative drug for intravenous high-dose AA therapy that improves the instability of AA. Intravenous high-dose AA is likely to be safe [18,19]. However, acute oxalate nephropathy has been reported in patients with renal failure who were given intravenous high-dose AA [20,21]. Therefore, patients with renal failure cannot receive intravenous high-dose AA therapy. Thus, development of stable ascorbic acid derivatives that exert strong antitumor activity at low doses is important. We have succeeded in developing two types of lipophilic derivatives of AA-2G, 6-*O*-acyl-2-*O*-α-d-glucopyranosyl-l-ascorbic acids having a straight-acyl chain of varying length from C_4_ to C_18_ (6-sAcyl-AA-2G, Figure 1) and a branched-acyl chain of varying length from C_6_ to C_16_ (6-bAcyl-AA-2G, Figure 1) [22,23]. 6-sAcyl-AA-2G and 6-bAcyl-AA-2G show efficient vitamin C activity after enzymatic hydrolysis to AA by α-glucosidase and lipase. Several of the 6-sAcyl-AA-2G derivatives showed higher skin permeability [22,23,24] and intestinal absorption [25] than those of AA-2G; that is, they are excellent provitamin C agents that can be efficiently absorbed and metabolized. More recently, we have reported that 6-sAcyl-AA-2G per se showed potent inhibitory activities of hyaluronidase and degranulation [26]. Of the 6-sAcyl-AA-2G derivatives, 6-sPalm-AA-2G, having a C_16_ straight-acyl chain, significantly suppressed passive cutaneous anaphylaxis reaction, which is a model of type I allergy. We are now searching for new pharmacological uses, not only use as an AA source, of 6-sAcyl-AA-2G and 6-bAcyl-AA-2G. The aim of the present study was to assess the possibility of using 6-sAcyl-AA-2G and 6-bAcyl-AA-2G as anti-cancer agents for infusion therapy instead of AA. We evaluated their effects on colon-26 (murine colon carcinoma) cells and in colon-26 tumor-bearing mice. 

## 2. Results and Discussion

**Figure 2:**
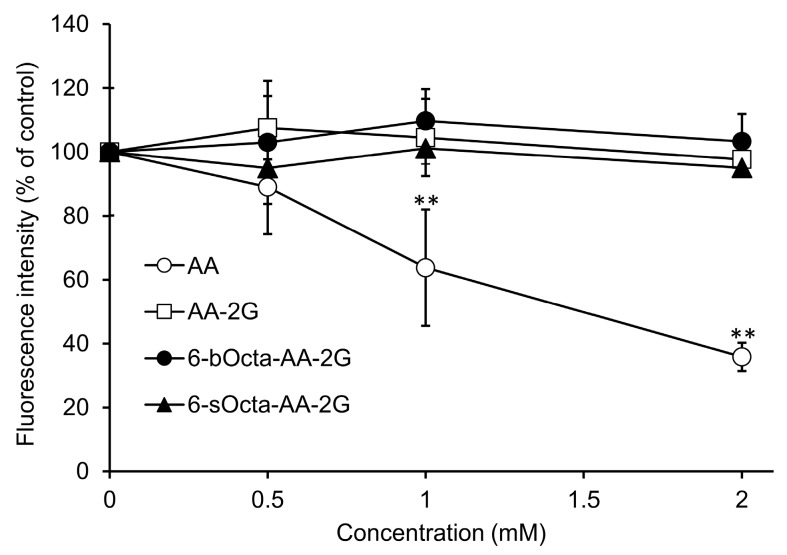
Cytotoxic activities of AA, 6-sOcta-AA-2G and 6-bOcta-AA-2G. Colon-26 cells were incubated in a medium containing each sample at indicated concentrations for 24 h. Vehicle-treated cells were arbitrarily set as 100% control viability. All data represent means ± SD of three independent cultures (** *p* < 0.01, compared with the control).

**Figure 3:**
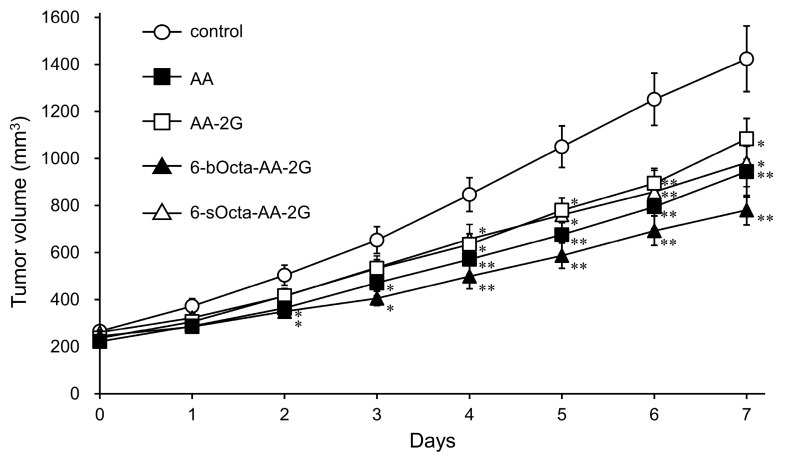
Tumor growth in colon-26 tumor-bearing CDF1 mice treated with AA and AA-2G (300 mg/kg) and with 6-sOcta-AA-2G and 6-bOcta-AA-2G (equimolecular amount of 30 mg/kg of AA). Tumor sizes were measured for 7 days during treatment. All data represent means ± SE, *n* = 16 (control, AA and AA-2G), *n* = 10 (6-sOcta-AA-2G) and *n* = 8 (6-bOcta-AA-2G). (* *p* < 0.05; ** *p* < 0.01, compared with the control).

Among the 6-sAcyl-AA-2G and 6-bAcyl-AA-2G derivatives, the antitumor activities of 6-sOcta-AA-2G and 6-bOcta-AA-2G having a relative long acyl chain and showing water solubility were evaluated [23]. Colon-26 cells were exposed to various concentrations of 6-sOcta-AA-2G and 6-bOcta-AA-2G. AA-2G, 6-sOcta-AA-2G and 6-bOcta-AA-2G did not show significant cytotoxic activity toward colon-26 even at a concentration of 2 mM, whereas AA exhibited cytotoxic activity in a concentration-dependent manner (Figure 2). It has been reported that 90% or more of AA-2G remained as the intact form in the presence of fibroblasts even after 60 h [16]. Thus, it is thought that the rates of hydrolysis of 6-sOcta-AA-2G and 6-bOcta-AA-2G were also slow and that sufficient AA to show cytotoxicity was not supplied to the medium. Our previous study showed that AA-2G had no cytotoxic activity in vitro but exhibited significant antitumor activity in vivo, that is, the antitumor activity of AA-2G requires a rapidly increase in AA concentration by rapid hydrolysis of AA-2G [17]. We then evaluated the antitumor activity by intravenous administration of 6-sOcta-AA-2G and 6-bOcta-AA-2G in tumor-bearing mice (Figure 3). AA and AA-2G were injected at 1.7 mmol/kg (equimolecular amount of 300 mg/kg of AA) and 6-sOcta-AA-2G and 6-bOcta-AA-2G were injected at 0.17 mmol/kg (equimolecular amount of 30 mg/kg of AA) on alternate days for 4 times. 6-sOcta-AA-2G and 6-bOcta-AA-2G significantly inhibited tumor growth despite the dose being 1/10 of the molar amount of AA and AA-2G unlike in the in vitro experiment. 6-bOcta-AA-2G tended to inhibit tumor growth more strongly than did AA, AA-2G and 6-sOcta-AA-2G. These results suggested that having suitable hydrophobicity and having branched acyl groups are important for the antitumor activity. 6-bOcta-AA-2G inhibited tumor growth more strongly than did AA despite the dose being 1/10 of the molar amount of AA. The results suggest that the antitumor activity of 6-bOcta-AA-2G was not merely an activity due to the supply of AA. 

**Figure 4:**
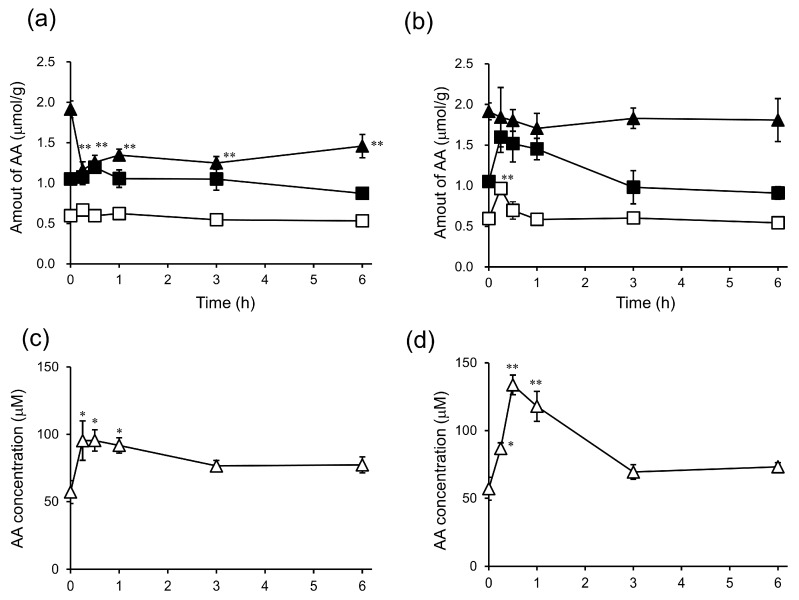
In vivo biodistribution and clearance of AA from 6-bOcta-AA-2G and 6-sOcta-AA-2G in colon-26 tumor-bearing CDF1 mice. 6-bOcta-AA-2G and 6-sOcta-AA-2G were injected intravenously into colon-26 tumor-bearing CDF1 mice. AA levels after administration of 6-bOcta-AA-2G in tissues (**a**) and in plasma (**c**) and AA levels after administration of 6-sOcta-AA-2G in tissues (**b**) and in plasma (**d**). Tumor (▲), liver (■), kidney (□) and plasma (Δ). All data represent means ± SE (*n* = 4). * *p* < 0.05; ** *p* < 0.01, compared with that at 0 min.

**Figure 5:**
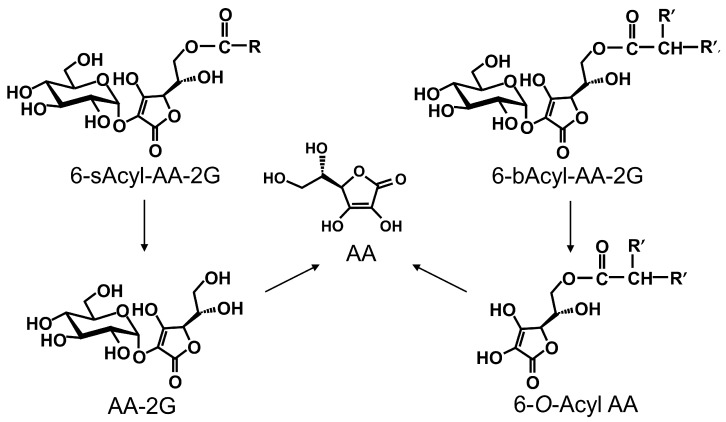
Metabolic pathways of 6-sAcyl-AA-2G and 6-bAcyl-AA-2G.

To reveal the active form of 6-bOcta-AA-2G, we studied the biodistribution and clearance of 6-bOcta-AA-2G and its metabolites in tumor-bearing mice and compared them with those of 6-sOcta-AA-2G and its metabolites. First, the contents of AA, a final metabolite from their derivatives, in tissues were measured. After administration of 6-bOcta-AA-2G, the AA level in the tumor was significantly decreased at 15 min, and the decreased level of AA did not recover even at 6 h (Figure 4a). Our previous studies showed that the AA content in the tumor was consumed to resist oxidative stress caused by reactive oxygen species (ROS) generated by high-dose intravenous administration of AA [17,27]. These results suggested that administration of 6-bOcta-AA-2G exerts oxidative stress on the tumor. The AA levels in the liver, kidney and plasma slightly increased (Figure 4a,c). The AA levels in the liver, kidney and plasma reached maximum value at 15 to 30 min after administration. After administration of 6-sOcta-AA-2G, the AA level in the tumor slightly decreased. The maximum decrease in the level of tumor AA occurred at 1 h after administration and the AA level had returned to the initial level at 3 h (Figure 4b). The magnitude of decrease in the amount of AA in the tumor was much smaller than that of 6-bOcta-AA-2G (Figure 4a,b). The AA levels in the liver, kidney and plasma greatly increased from the initial levels and reached maximum levels at 15 or 30 min (Figure 4b,d). The magnitude of increase in AA in the tissues by administration of 6-sOcta-AA-2G was larger than that of 6-bOcta-AA-2G. These results suggested that the magnitude of decrease in the amount of AA in the tumor after administration of 6-sOcta-AA-2G was smaller than that of 6-bOcta-AA-2G because 6-sOcta-AA-2G rapidly supplied much AA to each tissue. The metabolic pathways of 6-sAcyl-AA-2G and 6-bAcyl-AA-2G to AA depend on the structure of the acyl group in the molecule (Figure 5) [28]. In 6-sAcyl-AA-2G, the acyl group is firstly hydrolyzed by esterase to AA-2G, which is then hydrolyzed to AA by α-glucosidase. On the other hand, the acyl group of 6-bAcyl-AA-2G is hardly susceptible to hydrolysis by esterase due to its steric hindrance, and 6-bAcyl-AA-2G is metabolized to AA via 6-*O*-acyl ascorbic acid (6-*O*-Acyl AA). Thus, the release of AA from 6-bAcyl-AA-2G is slower than that from 6-sAcyl-AA-2G. The hypothesis is supported by the above findings. The results suggested that administration of 6-bOcta-AA-2G exerts greater oxidative stress on the tumor than does 6-sOcta-AA-2G and that the generation of AA from 6-bOcta-AA-2G was too slow for the amount of AA in the tumor to be recovered. Therefore, failure of the tumor to reduce oxidative stress may be one of the reasons that the antitumor activity of 6-bOcta-AA-2G was stronger than the antitumor activities of 6-sOcta-AA-2G, AA-2G and AA. 

**Figure 6:**
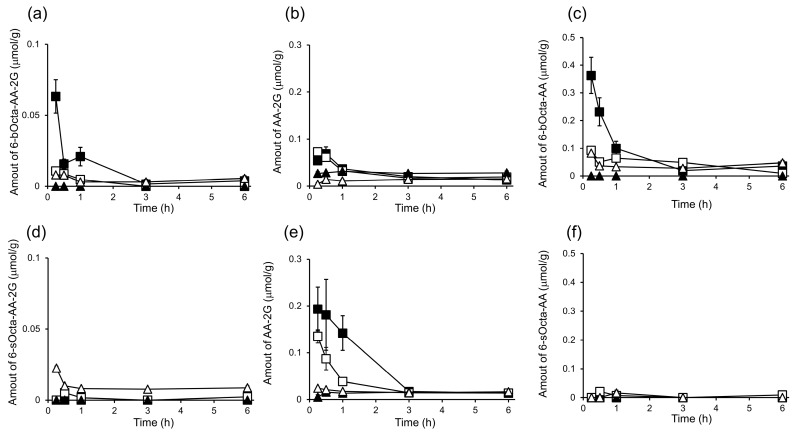
In vivo biodistribution and clearance of 6-bOcta-AA-2G, 6-sOcta-AA-2G and their two metabolites, AA-2G and AA, in colon-26 tumor-bearing CDF1 mice. 6-bOcta-AA-2G and 6-sOcta-AA-2G were injected intravenously into colon-26 tumor-bearing CDF1 mice. 6-bOcta-AA-2G levels (**a**), AA-2G levels (**b**) and 6-bOcta-AA levels (**c**) after administration of 6-bOcta-AA-2G. 6-sOcta-AA-2G levels (**d**), AA-2G levels (**e**) and 6-sOcta-AA levels (**f**) after administration of 6-sOcta-AA-2G. Tumor (▲), liver (■), kidney (□) and plasma (Δ). All data represent means ± SE (*n* = 4).

Next, the contents of 6-bOcta-AA-2G, 6-sOcta-AA-2G and their metabolites in the tissues were measured. 6-bOcta-AA-2G per se was detected in the liver, kidney and plasma, but the amounts of 6-bOcta-AA-2G in the kidney and plasma were small at 15 min after administration, and the levels of 6-bOcta-AA-2G were greatly decreased at 30 min (Figure 6a). The levels of AA-2G in the tissues were also low, and the levels were decreased at 1 h (Figure 6b). 6-*O*-(2-Propylpentanoyl)-l-ascorbic acid (6-bOcta-AA), a deglucosylated metabolite of 6-bOcta-AA-2G, was detected in the kidney, liver and plasma after 6-bOcta-AA-2G administration (Figure 6c). The levels of 6-bOcta-AA in the tissues were much higher than the levels of 6-bOcta-AA-2G at 15 min, suggesting that 6-bOcta-AA-2G was rapidly hydrolyzed to 6-bOcta-AA within 15 min. After administration of 6-sOcta-AA-2G, almost no 6-sOcta-AA-2G per se or 6-*O*-octanoyl-l-ascorbic acid (6-sOcta-AA), a deglucosylated metabolite of 6-sOcta-AA-2G, was detected in the tissues, while AA-2G was detected in the liver and the kidney (Figure 6d–f). The levels of AA-2G in the liver and the kidney were decreased at 3 h. These metabolic patterns are in agreement with our previous report as mentioned above. The results suggested that 6-bOcta-AA, a metabolite from 6-bOcta-AA-2G, seems to be involved in the strong antitumor effect of 6-bOcta-AA-2G.

**Figure 7:**
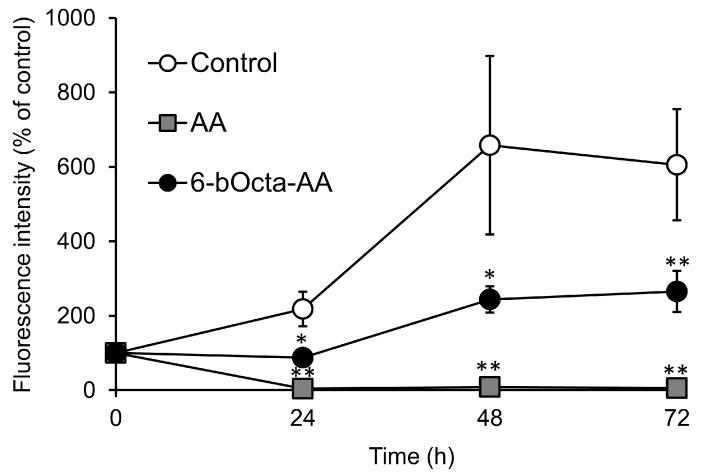
Cytotoxic activity of 6-bOcta-AA. Colon-26 cells were incubated in a medium containing each sample at 2 mM. Vehicle-treated cells were arbitrarily set as 100% control viability. All data represent means ± SD of three independent cultures (* *p* < 0.05; ** *p* < 0.01, compared with the control).

To clarify the involvement of 6-bOcta-AA in the antitumor activity, we synthesized 6-bOcta-AA and evaluated the effect of 6-bOcta-AA on colon-26 cells. Colon-26 cells were incubated with 6-bOcta-AA at 2 mM for 24, 48 and 72 h (Figure 7). 6-bOcta-AA showed significant cytotoxicity. AA showed cytotoxic activity in a short time (24 h), while 6-bOcta-AA sustainably inhibited cell proliferation (~72 h). These results suggested that the mechanism of the antitumor activity of 6-bOcta-AA is different from that of AA. The antitumor activity of AA is due to the pro-oxidant effect by its reducing activity as mentioned in the introduction. In the culture medium, AA is rapidly oxidized by a transition metal and converted to dehydroascorbic acid (DHA). 6-bOcta-AA, which has the same enediol lactone structure as that of AA, is also thought to be oxidized in a manner similar to that of AA. Recently, it was reported that DHA induces and increases endogenous oxidative stress in KRAS and BRAF mutant colorectal cancer cells via depletion of cell antioxidants and selectively kills the cancer cells [29]. Thus, the possibility that the oxidized form of 6-bOcta-AA shows antitumor activity was considered. However, since 6-bOcta-AA is oxidized in the culture period, it cannot be determined whether the antitumor activity of 6-bOcta-AA is due to the reduced form or the oxidized form.

**Figure 8:**
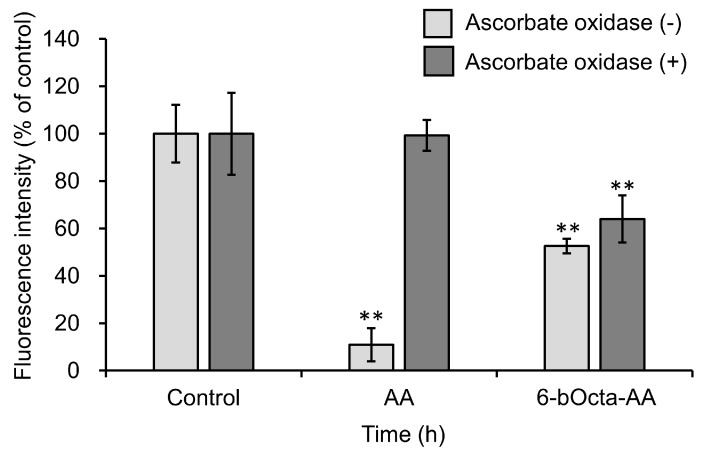
Cytotoxic activities of 6-bOcta-AA and ascorbate oxidase-treated 6-bOcta-AA. Colon-26 cells were incubated in a medium containing each sample at 4 mM for 1 h, and the cells were then washed and incubated for 48 h. Vehicle-treated cells were arbitrarily set as 100% control viability. All data represent means ± SD of three independent cultures (** *p* < 0.01, compared with the control).

To determine whether the antitumor activity of 6-bOcta-AA is due to 6-bOcta-AA per se and/or oxidized form of 6-bOcta-AA, the reduced form and the oxidized form of 6-bOcta-AA were respectively exposed to cells for a short time and the effects were compared. Oxidized forms of 6-bOcta-AA and AA were obtained by ascorbate oxidase treatment. Each reduced or oxidized form of 6-bOcta-AA and AA was added to a medium containing colon-26 cells and incubated for 1 h. The cells were then washed with PBS and incubated in a fresh medium for 48 h. The sample concentration optimized for short-term exposure was used (4 mM). The reduced form of AA showed significant cytotoxicity, but its activity was lost by treatment with ascorbate oxidase (Figure 8). In contrast, 6-bOcta-AA significantly inhibited cell proliferation in both treatments with ascorbate oxidase (−) and (+). These results suggested that the antitumor activity of 6-bOcta-AA-2G has a different point of action from that of AA and that the antitumor activity of 6-bOcta-AA-2G is due to both reduced and oxidized forms of 6-bOcta-AA, a metabolite of 6-bOcta-AA-2G.

We found in the present study that 6-bOcta-AA-2G had a strong inhibitory effect on tumor growth in tumor-bearing mice despite its dose being 1/10 of the molar amount of AA. Our results suggested that 6-bOcta-AA-2G itself has no antitumor activity but that 6-bOcta-AA released by hydrolysis of 6-bOcta-AA-2G gave the tumor large oxidative stress. In vivo biodistribution and clearance experiments showed that the slow supply of AA from 6-bOcta-AA-2G was one of the reasons for the strong antitumor activity of 6-bOcta-AA-2G and that the main active form with antitumor activity was 6-bOcta-AA. From the results of in vitro experiments, it became clear that 6-bOcta-AA showed cytotoxicity as both reduced and oxidized forms of 6-bOcta-AA. Although high-dose AA infusion therapy shows a high level of safety for many cancer patients, there is a problem that patients with renal failure cannot be treated. 6-bOcta-AA-2G, which showed strong antitumor activity at a lower dose than that of AA, can reduce the burden on the kidney by high-dose AA infusion therapy and would be useful instead of AA.

## 3. Materials and Methods

### 3.1. General Methods

6-bOcta-AA-2G and 6-sOcta-AA-2G were synthesized in our laboratory as described previously [22,23]. AA-2G was provided by Hayashibara Biochemical Laboratories (Okayama, Japan). Ascorbic acid, sodium ascorbate (AA-Na), heparin, calcein-AM solution and ascorbate oxidase from *Cucurbita* sp. were obtained from Wako Pure Chemical Industries (Osaka, Japan). Fetal bovine serum (FBS, heat-inactivated) was from Gibco (Waltham, MA, USA) and HyClone (Logan, UT, USA). RPMI-1640 medium and Triton X-100 were from Sigma-Aldrich (St. Louis, MO, USA). Penicillin and streptomycin solution and dithiothreitol (DTT) were purchased from Nacalai Tesque (Kyoto, Japan). 2-Propylvaleric acid and *n*-octanoic acid were obtained from Tokyo Chemical Industry (Tokyo, Japan). All of the chemicals used were of the highest grade commercially available. NMR spectra were obtained on a Varian NMR System 600 MHz instrument. The values of chemical shifts are expressed in ppm, and each coupling constant (J) is expressed in Hz. Electron spray ionization (ESI) high-resolution mass sptectra were recorded on a Bruker Daltonics MicrOTOF II instrument using direct sample injection. The purity of 6-bOcta-AA was assessed by HPLC and was found to be higher than 95%. HPLC analysis was performed on an Inertsil Ph-3 column ( 4.6 i.d. × 100 mm, 3 μm, GL Sciences Inc., Tokyo, Japan) kept at 40 °C with methanol/water/formic acid at 50:49.9:0.1 (*v*/*v*/*v*) at a flow rate of 0.7 mL/min. UV detection was performed at 254 nm.

### 3.2. Synthesis of 6-O-(2-Propylpentanoyl)-l-Ascorbic Acid

6-bOcta-AA was prepared according to the modified Tanaka’s method [30]. AA (1.00 g, 5.68 mmol) was dissolved in sulfric acid (11.8 mL) and 2-propylvaleric acid (0.9 mL, 5.68 mmol) was added. The mixture was stirred at room temperature for 24 h. The reaction mixture was then diluted with ice water (150 mL) and partitinoed twice with ethyl acetate (300 mL). The ethyl acetate layer was washed with saturated saline. After dehydration with sodium salfate, the filtrate was concentrated in vacuo. The resulting residue was chromatographed on a TOYOPEARL HW-40C column (3.0 i.d. × 35.0 cm) eluted with 250 mL of 40% methanol/water containing 0.5% formic acid and 250 mL of 60% methanol/water containing 0.5% formic acid to give 23 fractions. Fractions 17–21 (1.28 g) were then recrystallized twice from ethyl acetate/isopropyl ether to give 6-bOcta-AA (0.26 g, yield 15.1%). ^1^H NMR (CD_3_OD, 600 MHz) δ_H_: 0.91 (6H, t, *J* = 7.2 Hz), 1.31 (4H, m), 1.45 (2H, m), 1.60 (2H, m), 2.44 (1H, m), 4.07 (1H, ddd, *J* = 1.8, 6.6, 7.2 Hz, 5-H), 4.19 (1H, dd, *J* = 6.6, 11.4 Hz, 6-Ha), 4.24 (1H, dd, *J* = 7.2, 11.4 Hz, 6-Hb), 4.71 (1H, d, *J* = 1.8 Hz, 4-H). ^13^C-NMR (CD_3_OD, 150 MHz): δ_C_ 12.88 (×2), 20.23 (×2), 34.39, 34.42, 45.08, 64.24, 66.68, 75.77, 118.67, 152.51, 171.69, 176.39. ^1^H-NMR and ^13^C-NMR spectrum data are shown in Supplementary Materials (Figures S1 and S2). ESI-HRMS [M–H]^−^: calcd. for C_14_H_21_O_7_: 301.1302, found 301.1293. HPLC: rt 7.59 min, 96.2% purity.

### 3.3. Cell Lines

Murine colon carcinoma (colon-26) cells were purchased from RIKEN BRC CELL BANK (Tsukuba, Japan). Colon-26 cells were grown in RPMI 1640 medium supplemented with 10% FBS, 100 units/mL penicillin and 100 μg/mL streptomycin at 37 °C in 5% CO_2_.

### 3.4. Evaluation of Cytotoxic Activity

The cells were suspended in 10% FBS/RPMI 1640 medium and seeded in a 96-well microplate at a density of 1.0 × 10^4^ cells/100 μL/well and then incubated for 24 h at 37 °C in 5% CO_2_. After incubation, each medium was replaced with 90 μL of fresh medium and then 10 μL of each sample dissolved in the medium was added and the cells were incubated for 24 h. When 72-h incubation experiments were carried out, the cells were seeded in a 96-well microplate at a density of 3.0 × 10^3^ cells/100 μL/well. The cells were then washed with 100 μL of PBS (−), and 100 μL of calcein-AM solution (5 μM) was added. After 30 min of incubation, 20 μL of 0.6% Triton X-100 solution was added to lyse the cells. The fluorescence intensity (FI) of the cell lysate was recorded on a microplate reader (Ex. 485 nm, Em. 527 nm, Varioskan Flash from Thermo Scientific, Waltham, MA, USA). Cell viability (%) was caluculated as (FI of treated-FI of blank)/(FI of control-FI of blank) × 100. The difference in cell viability between the control and treatment was analyzed by Dunnett’s test (** *p* < 0.01).

### 3.5. Evaluation of Cytotoxic Activity in Short-Term Exposure of 6-bOcta-AA and AA

The cells were suspended in 10% FBS/RPMI 1640 medium and seeded in a 96-well microplate at a density of 3.0 × 10^3^ cells/100 μL/well and then incubated for 24 h at 37 °C in 5% CO_2_. After incubation, each medium was replaced with 100 μL of each sample. Oxidized 6-bOcta-AA and AA were prepared to incubate 6-bOcta-AA and AA in the medium containing 5.0 U/mL of ascorbate oxidase for 1 h at 37 °C just before use. After 1 h of incubation, the cells were washed with a fresh medium twice and incubated for 48 h. The cytotoxicity activity was evaluated as described in the above section. 

### 3.6. Evaluation of Antitumor Activity in Tumor-Bearing Mice

Balb/c mice were purchased from CLEA Japan (Tokyo, Japan) and maintained at a room temperature of 20 ± 5 °C. Colon-26 cells were implanted at a density of 1.0 × 10^6^ cells in five-week-old female Balb/c mice subcutaneously in the left and right dorsal areas. When transplanted, tumor sizes were more than 1 cm in diameter, the tumors were excised and small pieces of the tumor (approximately 2-mm cubes) were engrafted subcutaneously into the left dorsal area of each five-week-old male CDF1 mouse (Japan SLC, Shizuoka, Japan). When tumor sizes had reached 8–10 mm in diameter after implantation, the mice were used for studies of antitumor activity, biodistribution and clearance. AA-Na and AA-2G were dissolved in PBS (−) at a molar equivalent of 300 mg/kg of AA (1.7 mmol/kg). 6-sOcta-AA-2G and 6-bOcta-AA-2G were dissolved in PBS (−) at a molar equivalent of 30 mg/kg of AA (0.17 mmol/kg). The pH values of AA-2G, 6-sOcta-AA-2G and 6-bOcta-AA-2G were adjusted to neutral by adding sodium hydroxide. The solutions were then injected intravenously 4 times into colon-26 tumor-bearing mice on alternate days. Tumor size was calculated from caliper measurements using volume = (length) × (width)^2^ × 0.5 [31]. The differences in tumor size between the treatment groups and control group were analyzed by Dunnett’s test (* *p* < 0.05, ** *p* < 0.01). The experiments were approved by the Committee for Ethics in Animal Experiments of the Prefectural University of Hiroshima (permit number: 13SA005; approval date: 25 June 2013 and permit number: 15SA006; approval date: 25 May 2015).

### 3.7. In Vivo Biodistribution and Clearance of 6-bOcta-AA-2G, 6-sOcta-AA-2G and Their Metabolites

The mice that had been injected intravenously with 6-bOcta-AA-2G or 6-sOcta-AA-2G were sacrificed under anesthesia by isoflurane. The liver, kidney, tumor and blood were collected at 15, 30, 60, 180 and 360 min after injection. A blood sample was collected by heart puncture into a heparin-coated syringe. Plasma was separated from whole blood by centrifugation at 10,000× *g* for 10 min at 4 °C. The samples were stored at −80 °C until analysis. Samples were homogenized with 85% acetonitrile including 250 mg/L of DTT (100 mg wet tissue or 100 μL plasma/400 μL solution). Then, all of the samples were centrifuged at 10,000× *g* for 10 min at 4 °C and the supernatants were collected. The supernatants were analyzed by HPLC. Separation of AA-2G and AA was achieved by isocratic elution of an HILIC column (4.6 i.d. × 250 mm, 5 μm, GL Sciences Inc., Tokyo, Japan) kept at 40 °C with 85% acetonitrile/66.7 mM ammonium acetate at a flow rate of 0.7 mL/min [32]. Separation of 6-bOcta-AA-2G, 6-sOcta-AA-2G and 6-*O*-Acyl AA was achieved by isocratic elution of an Inertsil Ph column (4.6 i.d. × 250 mm, 5 μm, GL Sciences Inc., Tokyo, Japan) kept at 40 °C with 55% methanol containing 0.5% formic acid at a flow rate of 0.7 mL/min. The absorbance at 240 nm was monitored. The mean concentration ± standard error of the mean (SE) was calculated for each time point. The difference at each time point between the initial value (0 min) and treatment groups was analyzed by Dunnett’s test (* *p* < 0.05; ** *p* < 0.01).

## 4. Conclusions

6-bOcta-AA-2G is an excellent provitamin C agent. Intravenous administration of 6-bOcta-AA-2G suppressed tumor growth in colon-26 tumor-bearing mice more strongly than did AA at 1/10 of the molar amount of AA. The antitumor effect of 6-bOcta-AA-2G was not due to itself but was due to that 6-bOcta-AA released by hydrolysis of 6-bOcta-AA-2G causing injury to the tumor. In vivo biodistribution and clearance experiments showed that slow supply of AA from 6-bOcta-AA-2G was one of the reasons for the strong antitumor activity of 6-bOcta-AA-2G and that the main active form with antitumor activity was 6-bOcta-AA. In vitro experiments showed that 6-bOcta-AA exhibited cytotoxicity as both reduced and oxidized forms. Our results provide evidence that 6-bOcta-AA-2G is a potent candidate as an alternative drug to intravenous high-dose AA.

## Supplementary Materials

Supplementary materials can be found at http://www.mdpi.com/1422-0067/19/2/535/s1.

## References

[1] Du J., Cullen J.J., Buettner G.R. (2012). Ascorbic acid: Chemistry, biology and the treatment of cancer. Biochim. Biophys. Acta.

[2] Cameron E., Pauling L. (1978). Supplemental ascorbate in the supportive treatment of cancer: Reevaluation of prolongation of survival times in terminal human cancer. Proc. Natl. Acad. Sci. USA.

[3] Hoffer L.J., Levine M., Assouline S., Melnychuk D., Padayatty S.J., Rosadiuk K., Rousseau C., Robitaille L., Miller W.H. (2008). Phase I clinical trial of IV ascorbic acid in advanced malignancy. Ann. Oncol..

[4] Parrow N.L., Leshin J.A., Levine M. (2013). Parenteral ascorbate as a cancer therapeutic: A reassessment based on pharmacokinetics. Antioxid. Redox Signal..

[5] Padayatty S.J., Sun H., Wang Y., Riordan H.D., Hewitt S.M., Katz A., Wesley R.A., Levine M. (2004). Vitamin C pharmacokinetics: Implications for oral and intravenous use. Ann. Intern. Med..

[6] Chen Q., Espey M.G., Krishna M.C., Mitchell J.B., Corpe C.P., Buettner G.R., Shacter E., Levine M. (2005). Pharmacologic ascorbic acid concentrations selectively kill cancer cells: Action as a pro-drug to deliver hydrogen peroxide to tissues. Proc. Natl. Acad. Sci. USA.

[7] Chen Q., Espey M.G., Sun A.Y., Lee J.H., Krishna M.C., Shacter E., Choyke P.L., Pooput C., Kirk K.L., Buettner G.R. (2007). Ascorbate in pharmacologic concentrations selectively generates ascorbate radical and hydrogen peroxide in extracellular fluid in vivo. Proc. Natl. Acad. Sci. USA.

[8] Chen Q., Espey M.G., Sun A.Y., Pooput C., Kirk K.L., Krishna M.C., Khosh D.B., Drisko J., Levine M. (2008). Pharmacologic doses of ascorbate act as a prooxidant and decrease growth of aggressive tumor xenografts in mice. Proc. Natl. Acad. Sci. USA.

[9] Oberley T.D., Oberley L.W. (1997). Antioxidant enzyme levels in cancer. Histol. Histopathol..

[10] Cimmino L., Dolgalev I., Wang Y., Yoshimi A., Martin G.H., Wang J., Ng V., Xia B., Witkowski M.T., Mitchell-Flack M. (2017). Restoration of TET2 function blockes aberrant self-renewal and leukemia progression. Cell.

[11] Agathocleous M., Meacham C.E., Burgess R.J., Piskounova E., Zhao Z., Crane G.M., Cowin B.L., Bruner E., Murphy M.M., Chen W. (2017). Ascorbate regulates haematopoietic stem cell function and leukaemogenesis. Nature.

[12] Ma Y., Chapman J., Levine M., Polireddy K., Drisko J., Chen Q. (2014). High-dose parenteral ascorbate enhanced chemosensitivity of ovarian cancer and reduced toxicity of chemotherapy. Sci. Transl. Med..

[13] Yamamoto I., Tanaka M., Muto N. (1993). Enhancement of in vitro antibody production of murine splenocytes by ascorbic acid 2-*O*-α-glucoside. Int. J. Immunopharmacol..

[14] Ichiyama K., Mitsuzumi H., Zhong M., Tai A., Tsuchioka A., Kawai S., Yamamoto I., Gohda E. (2009). Promotion of IL-4-and IL-5-dependent differentiation of anti-μ-primed B cells by ascorbic acid 2-glucoside. Immunol. Lett..

[15] Yamamoto I., Suga S., Mitoh Y., Tanaka M., Muto N. (1990). Antiscorbutic activity of l-ascorbic acid 2-glucoside and its availability as a vitamin C supplement in normal rats and guinea pigs. J. Pharmacobiodyn..

[16] Yamamoto I., Muto N., Murakami K., Akiyama J. (1992). Collagen synthesis in human skin fibroblasts is stimulated by a stable form of ascorbate, 2-*O*-α-d-glucopyranosyl-l-ascorbic acid. J. Nutr..

[17] Miura K., Tai A. (2017). 2-*O*-α-d-Glucopyranosyl-l-ascorbic acid as an antitumor agent for infusion therapy. Biochem. Biophys. Rep..

[18] Padayatty S.J., Sun A.Y., Chen Q., Espey M.G., Drisko J., Levine M. (2010). Vitamin C: Intravenous use by complementary and alternative medicine practitioners and adverse effects. PLoS ONE.

[19] Stephenson C.M., Levin R.D., Spector T., Lis C.G. (2013). Phase I clinical trial to evaluate the safety, tolerability, and pharmacokinetics of high-dose intravenous ascorbic acid in patients with advanced cancer. Cancer Chemother. Pharmacol..

[20] Levine M., Rumsey S.C., Daruwala R., Park J.B., Wang Y. (1999). Criteria and recommendations for vitamin C intake. JAMA.

[21] McAllister C.J., Scowden E.B., Dewberry F.L., Richman A. (1984). Renal failure secondary to massive infusion of vitamin C. JAMA.

[22] Yamamoto I., Tai A., Fujinami Y., Sasaki K., Okazaki S. (2002). Synthesis and characterization of a series of novel monoacylated ascorbic acid derivatives, 6-*O*-acyl-2-*O*-α-d-glucopyranosyl-l-ascorbic acids, as skin antioxidants. J. Med. Chem..

[23] Tai A., Kawasaki D., Sasaki K., Gohda E., Yamamoto I. (2003). Synthesis and characterization of 6-*O*-acyl-2-*O*-α-d-glucopyranosyl-l-ascorbic acids with a branched-acyl chain. Chem. Pharm. Bull..

[24] Tai A., Goto A., Ishiguro Y., Suzuki K., Nitoda T., Yamamoto I. (2004). Permeation and metabolism of a series of novel lipophilic ascorbic acid derivatives, 6-*O*-acyl-2-*O*-α-d-glucopyranosyl-l-ascorbic acids with a branched-acyl chain, in a human living skin equivalent model. Bioorg. Med. Chem. Lett..

[25] Tai A., Fujinami Y., Matsumoto K., Kawasaki D., Yamamoto I. (2002). Bioavailability of a series of novel acylated ascorbic acid derivatives, 6-*O*-acyl-2-*O*-α-d-glucopyranosyl-l-ascorbic acids, as an ascorbic acid supplement in rats and guinea pigs. Biosci. Biotechnol. Biochem..

[26] Miura K., Morishita Y., Matsuno H., Aota Y., Ito H., Tai A. (2017). Anti-allergic activity of monoacylated ascorbic acid 2-glucosides. Molecules.

[27] Miura K., Yazama F., Tai A. (2015). Oxidative stress-mediated antitumor activity of erythorbic acid in high doses. Biochem. Biophys. Rep..

[28] Tai A., Kawasaki D., Goto S., Gohda E., Yamamoto I. (2003). Vitamin C activity in guinea pigs of 6-*O*-acyl-2-*O*-α-d-glucopyranosyl-l-ascorbic acids with a branched-acyl chain. Biosci. Biotechnol. Biochem..

[29] Yun J., Mullarky E., Lu C., Bosch K.N., Kavalier A., Rivera K., Roper J., Chio I.I.C., Giannopoulou E.G., Rago C. (2015). Vitamin C selectively kills KRAS and BRAF mutant colorectal cancer cells by targeting GAPDH. Science.

[30] Tanaka H., Yamamoto R. (1966). Synthesis of esters of ascorbic acid and their physicochemical properties. Yakugaku Zasshi.

[31] Haranaka K. (1984). Antitumor activity of murine tumor necrosis factor (TNF) against transplanted murine tumors and heterotransplanted human tumors in nude mice. Int. J. Cancer.

[32] Tai A., Gohda E. (2007). Determination of ascorbic acid and its related compounds in foods and beverages by hydrophilic interaction liquid chromatography. J. Chromatogr. B Analyt. Technol. Biomed. Life Sci..

