# Supplementary material for: Potential Antitumor Activity of 2-O-α-d-Glucopyranosyl-6-O-(2-Pentylheptanoyl)-l-Ascorbic Acid
Source: Int J Mol Sci. 2018 Feb 10;19(2):535. doi: 10.3390/ijms19020535 (PMC5855757; doi:10.3390/ijms19020535)
Supplement: Supplementary file 1 [file ijms-19-00535-s001.pdf]

Supplementary materials

Antitumor activity of 2-*O*-α-D-glucopyranosyl-6-*O*-(2-pentylheptanoyl)-L-ascorbic acid

Kaori Miura [1], Misaki Haraguchi [1], Hideyuki Ito [2] and Akihiro Tai [1,*]

[1] Faculty of Life and Environmental Sciences, Prefectural University of Hiroshima, 5562 Nanatsuka-cho, Shobara, Hiroshima 727-0023, Japan; q531005dd@ed.pu-hiroshima.ac.jp (K.M.); @ed.pu-hiroshima.ac.jp (M.H.); hito@fhw.oka-pu.ac.jp (H.I.)

[2] Faculty of Health and Welfare Science, Okayama Prefectural University, 111 Kuboki, Soja, Okayama 719-1197, Japan; hito@fhw.oka-pu.ac.jp

* Correspondence: atai@pu-hiroshima.ac.jp; Tel.: +81-824-74-1779

^{1}H-NMR (CD_3OD, 600 MHz)

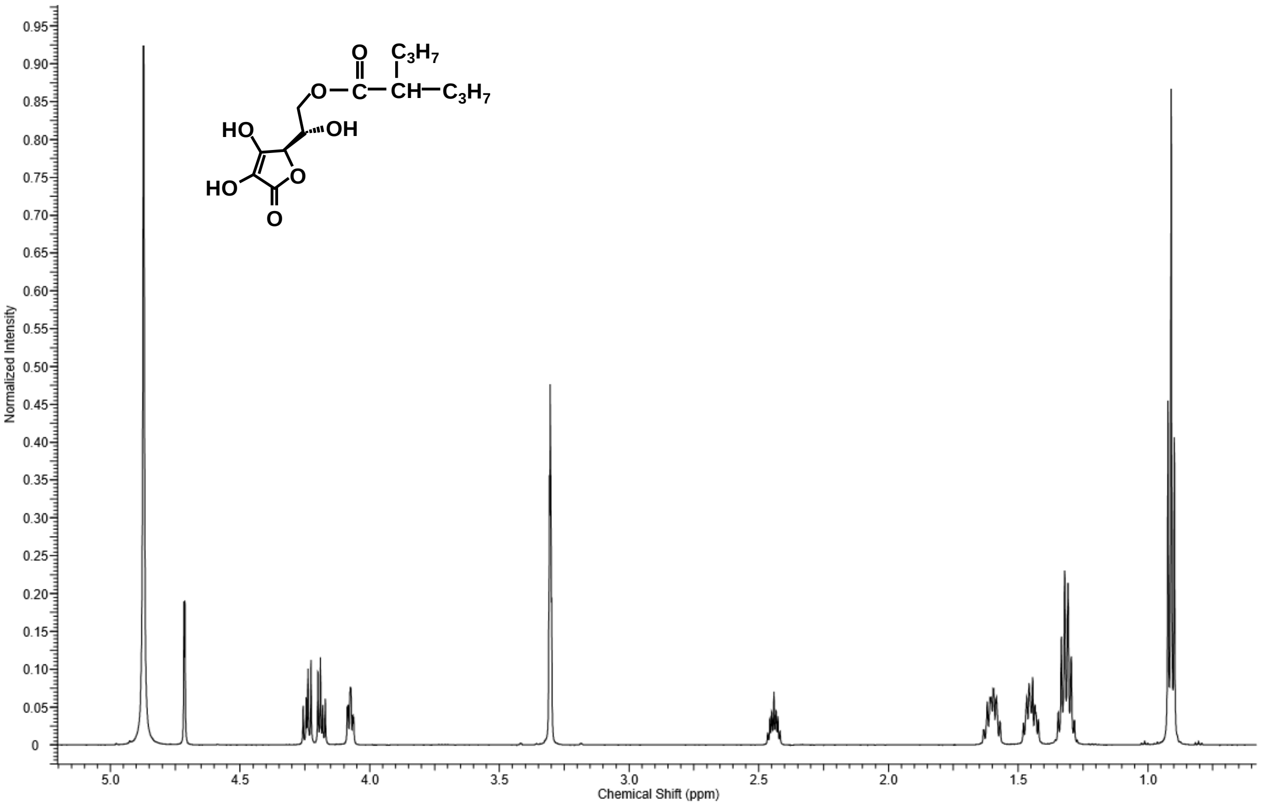

Figure S1. ^{1}H-NMR spectrum of 6-bOcta-AA

^{13}C-NMR (CD_3OD, 150 MHz)

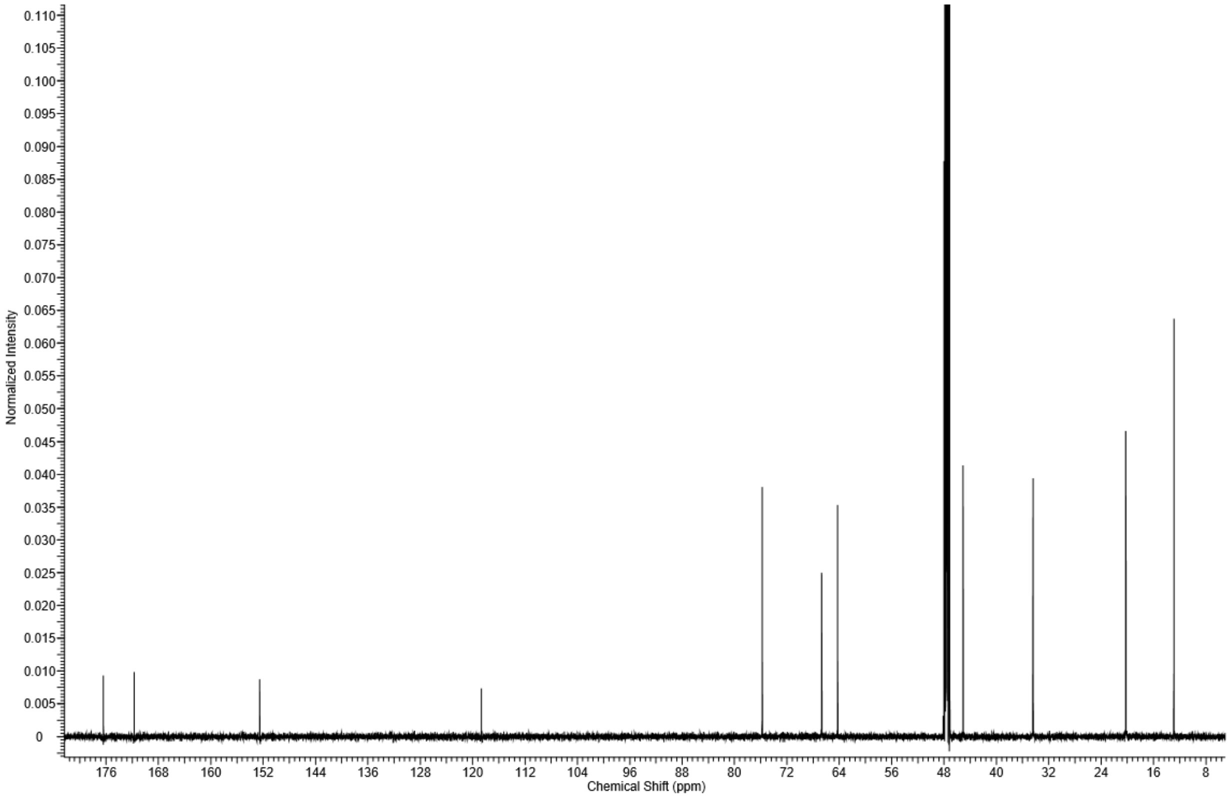

Figure S2. ^{13}C-NMR spectrum of 6-bOcta-AA
